# The Effect of Thyroid Function on GDF15 Levels

**DOI:** 10.3390/ijms262211073

**Published:** 2025-11-16

**Authors:** Nicia I. Profili, Edoardo Fiorillo, Valeria Orrù, Francesco Cucca, Alessandro P. Delitala

**Affiliations:** 1 Department of Medicine, Surgery, and Pharmacy, University of Sassari, 07100 Sassari, Italy; 2 Institute for Genetic and Biomedical Research, National Research Council, 08045 Lanusei, Italy; edoardo.fiorillo@irgb.cnr.it (E.F.); valeria.orru@irgb.cnr.it (V.O.); fcucca@uniss.it (F.C.); 3 Department of Biomedical Science, University of Sassari, 07100 Sassari, Italy

**Keywords:** GDF-15, thyroid hormone, TSH, cardiovascular disease, thyrotropin

## Abstract

Growth differentiation factor 15 (GDF15) is a stress-response cytokine, which exerts different actions in physiological and pathological conditions. Thyroid disorders are common in the general population and their role on GDF15 levels has not been sufficiently addressed. Serum levels of GDF-15 and thyroid function were assessed in a large sample from the SardiNIA cohort (n = 4413). We further collected antibodies against thyroperoxidase and anti-thyroglobulin in all participants. Thyroid function correlated with GDF15. Specifically, after adjusting for covariates, thyrotropin had a positive association with GDF15, while free thyroxine was negatively correlated. We also found that subjects with circulating antibodies against thyroperoxidase had lower GDF15 levels. The reduced level of the thyroid hormone can cause a decreased metabolic activity and higher cell stress response, which, in turn, increases the production of GDF-15 as a defense mechanism. The association between antibodies against thyroperoxidase and GDF15 deserves additional study to elucidate the pathophysiologic relationship.

## 1. Introduction

Cardiovascular disease is the leading cause of death worldwide [1] and its association with thyroid hormone disorders is well documented [2]. This correlation is well documented in the case of overt disorders, defined by the presence of impaired thyrotropin (TSH) and free thyroxine (FT4). Indeed, overt hypothyroidism (i.e., increased TSH and reduced FT4) is associated with hypertension and diastolic dysfunction and can further reduce stroke volume and cardiac contractility [3]. In addition, patients with overt hypothyroidism can develop sinus bradycardia, atrio-ventricular block, and pericardial effusion [4]. The deleterious effect of overt hypothyroidism on the cardiovascular system is also extended to lipid metabolism. Indeed, a great body of evidence agrees that low thyroid function is associated with a worse lipid profile and, therefore, with endothelial dysfunction [4]. On the other hand, overt hypothyroidism (i.e., suppressed TSH and increased FT4) is associated with an increased frequency of specific arrhythmias, such as atrial fibrillation and atrial flutter [5], which increases the risk of ischemic stroke. In addition, patients with overt hyperthyroidism may develop systolic hypertension, increased left ventricular mass, and high-output heart failure [6].

The role of subclinical disorders (impaired TSH and FT4 within the reference range) on cardiovascular disease remains controversial. Subclinical hypothyroidism was linked to increased thickness of carotid intima-media in some studies [7], but not in other [8]. This possible association is somewhat based on the finding that a mild elevation of TSH can increase LDL concentration [4]. Studies also reported that patients with TSH ≥ 10 mUI/mL also had impaired diastolic function [9] and increased aortic stiffness [10], which is a predictor of mortality in patients with cardiovascular disease. On the other hand, subclinical hyperthyroidism (e.g., reduced TSH and FT4 within the reference range) increased the risk to develop of atrial fibrillation and mortality [11].

Human growth differentiation factor 15 (GDF15) is a member of the transforming growth factor-β cytokine superfamily, a widely distributed protein which has different roles in physiological as well as pathological conditions [12]. The cells which play roles in the inflammatory process express GDF15 receptors [13]. Levels of GDF15 dramatically increase when disease develops [14]. Indeed, GDF15 secretion is stimulated by hypoxia, chronic inflammatory disease, chronic kidney, and liver disease [7]. GDF15 has a weak expression in healthy cardiovascular tissue but it dramatically increases in response to specific cardiovascular disease. Indeed, in hypertensive patients, GDF15 was positively associated with the thickness of posterior wall of the left ventricle and left ventricular mass [15] and treatment with recombinant GDF15 leads to a decrease in hypertrophy and reduced ventricular dilation in heart failure mice [16]. Furthermore, higher GDF15 levels are associated with the development of heart failure, acute coronary syndrome, and atrial fibrillation [17,18], and also emerged to be predictive of adverse outcomes in patients with cardiovascular disease [19]. Recent data also demonstrated the role of GDF-15 on the endocrine system, which displayed pleiotropic effects. Specifically, the role of GDF-15 has been evaluated at the thyroid level due to its common effects on the body’s energy metabolism and on the regulation of energy substrate utilization. In this study, we tested the possible association between GDF-15 and thyroid function.

## 2. Results

Summary statistics of the parameters and participants considered are reported in Table 1.

Females were younger (47.4 vs. 49.0, *p* < 0.05) and had lower BMI (24.0 vs. 26.6, *p* < 0.001) compared to males which had lower TSH and GDF15 values (1.40 vs. 1.71, *p* < 0.001 and 204.4 vs. 202.7, *p* < 0.001, respectively). Males also had a higher frequency of diabetes (5.0% vs. 3.7%, *p* < 0.05), hypertension (3.3% vs. 28.1%, *p* < 0.001), and were more likely to be smokers (24.2% vs. 14.3%, *p* < 0.001) than females. The frequency of TPOAb and TGAb were both higher in females compared to males (19.0 vs. 10.1% *p* < 0.001 and 12.1% vs. 5.9% *p* < 0.001, respectively).

**Figure 1:**
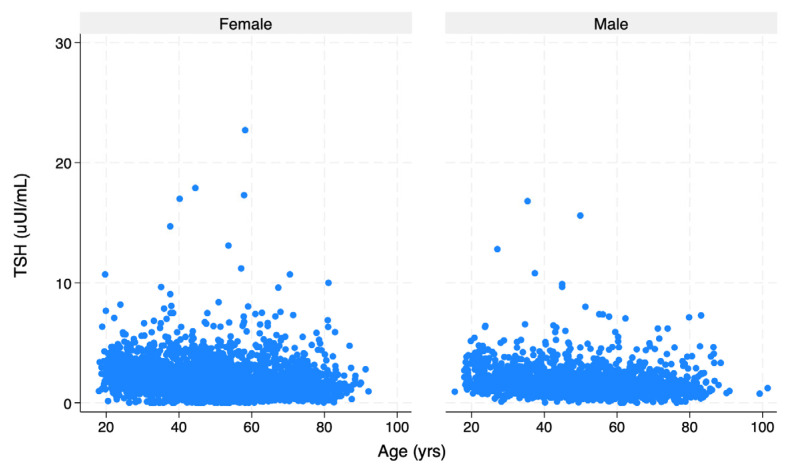
Distribution of TSH across age in men and women. Abbreviation: TSH, thyrotropin.

**Figure 2:**
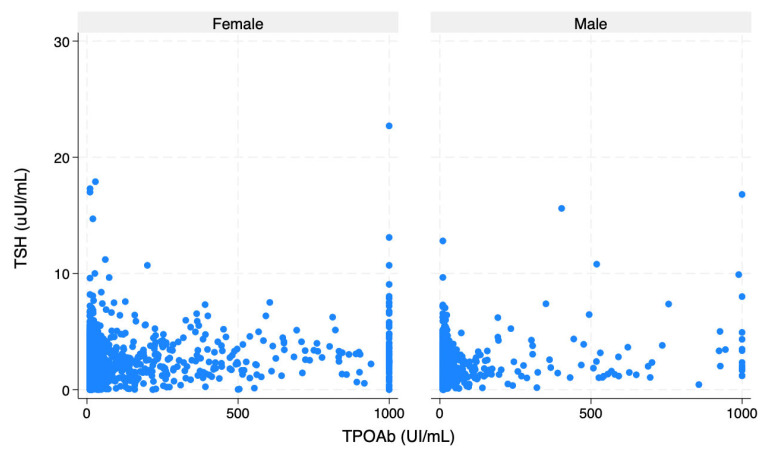
Distribution of TSH across TPOAb in men and women. Abbreviations: TSH, thyrotropin; TPOAb, antibodies against thyroperoxidase.

Figure 1 shows the TSH distribution across age, stratified by gender. Figure 2 depicts the correlation between TSH and TPOAb in men and women.

Table 2 describes the results of univariate analysis. Age, BMI, HbA1c, TSH, FT4, TPOAb, whether they smoke, and lipid parameters were all associated with GDF15 (0.029 or lower).

Table 3 shows the variables associated with GDF15. Age, TSH, history of diabetes, hypertension, and smoking positively correlated with GDF15, while FT4 and total cholesterol showed a negative association with GDF15.

Table 4 shows the median concentration of GDF15 across patients with positivity for TPOAb, TGAb, or both. Antibodies were further stratified into two different groups: standard cut-off (TPOAb ≥ 35 IU/mL and TGAb > 40 UI/mL) and high cut-off (TPOAb and TGAb > 100 UI/mL). Due to the correlation between GDF15 and age, we split the sample according to the median age and the results reported in Table 4 are limited to subjects aged 48 yrs or older. GDF15 levels were lower in participants who were positive for TPOAb, both for standard (*p* = 0.037) and high values cut-off (*p* = 0.002)

## 3. Discussion

In this study, we reported a positive association between GDF15 and TSH. Specifically, GDF15 linearly increased along with TSH value, after adjusting for covariates. Consistently, FT4 had a negative correlation with GDF15. Hypothyroidism, defined as an increased THS value, is a well-known cause of cardiovascular disease, which acts at different sites. Indeed, hypothyroidism has an impact on lipid parameters, which is documented in the case of overt hypothyroidism (increased TSH and reduced FT4) [20] and is less clear in case of subclinical hypothyroidism [21] (increased TSH and normal FT4). Menopause has been reported as a possible confounder of this association [22]. Similarly, high TSH is described to increase the risk of atherosclerosis, in particular in the case of overt hypothyroidism [20]. However, the role of subclinical hypothyroidism in this context still needs to be elucidated [8].

GDF15 is expressed in nearly all tissues and modulates different pathways: inflammatory, apoptosis, and angiogenesis [23]; however, the exact biological function is still not understood. Some studies also showed that GDF15 may have opposing functions which makes the interpretation of its role in the cardiovascular system extremely puzzling [23]. GDF15 was also reported as prognostic biomarker in patients with acute coronary syndrome [24]. Other studies also reported the results of a variation in GDF15 in specific patients. For example, the GUSTO-IV trial analyzed the role of GDF15 in patients with NON-ST-elevation acute coronary syndrome (NSTE-ACS), a clinical condition characterized by episodes of chest pain at rest of with minimal exertion and whose presence is associated with higher mortality. Increasing tertiles of GDF15 were correlated with higher risk of death within 1 year, particularly in patients with values ≥1808 ng/L [25]. The FRISC II study showed that GDF15 may improve risk stratification in NSTE-ACS patients [24] and demonstrated that elevated levels of GDF15 independently predicted risk of death or recurrent myocardial infarction in patients not treated with revascularization. Finally, the analysis from the PLATO study revealed that higher GDF15 levels were associated with increased risk of stroke, myocardial infarction, and mortality [26], thus confirming previous reports. Interestingly, GDF15 was also associated with lower left ventricular ejection fraction and impaired diastolic function [27]. GDF15 expression has been reported to be increased in ischemia–reperfusion injuries. Indeed, GDF15-deficient mice had increased infarct size and cardiomyocyte apoptosis REF 130; furthermore, the mortality after infarction was greater in Gdf15-knockout mice compared to wild-type mice due to greater rupture of left ventricular free wall and higher occurrence of hemothorax [16]. On the contrary, mice treated with recombinant GDF15 showed an antiapoptotic effect on cultured cardiomyocytes in mice [28]. The role of GDF15 in atherosclerosis is not completely understood. Indeed, GDF15 was originally found to be overexpressed in activated macrophages [29] and its expression is also increased during advanced atherosclerotic processes in mice [30]. Studies supporting the role of GDF15 in the atherosclerotic process also showed that mice with a combined GDF15 and apolipoprotein E (ApoE) deficiency had reduced macrophage activation in atherosclerotic lesions [31]. Another study in LDL-knockout mice reported that GDF15 promoted early atherogenesis [30]. However, other studies reported a protective effect on atherogenesis. Indeed, de Jager et al. demonstrated that a deficiency of GDF15 impaired macrophage migration and, therefore, improved the stability of atherosclerotic plaque [30]. Further, APOE (−/−) mice with GDF15 overexpression had a smaller atherosclerotic plaque [32]. Finally, studies also showed that GDF15 may inhibit platelet integrin activation, thus preventing thrombus formation [33]. Overall, these contradictory results might be explained by methodological differences.

The association between GDF15 and thyroid disorders is less clear. The study by Arslan et al. showed that, despite comparable levels of GDF15, children with subclinical hypothyroidism had diastolic dysfunction compared to healthy controls [34]. Another study reported that GDF15 levels were increased in hyperthyroid patients compared to euthyroid subjects [35]. The authors also showed that treatment of hyperthyroidism reduced GDF15 values. To strengthen their findings, they also injected thyroid hormones into mice and observed an increased expression of GDF15 in their brown adipose tissue. These findings deserve a specific evaluation. Indeed, a study by Arslan et al. described a group of pediatric patients in which the role of subclinical hypothyroidism at cardiovascular level was weak and GDF15 was commonly found to be increased during aging [34]. On the other hand, the study by Zhao et al. did not specify the presence of cardiovascular disease in hyperthyroid patients and the analysis was limited to a very small sample of patients. Univariate analysis showed a negative association between TSH and GDF15, thus suggesting higher values, but the multivariate regression analysis revealed a positive association between TSH and GDF15. A major role in this apparent contradictory result is played by age. Subjects with reduced TSH were older compared to those with euthyroid and hypothyroid and the regression model showed that age was a key determinant of GDF15. Overall, we can speculate that the reduced level of thyroid hormones can cause decreased metabolic activity and higher cell stress responses. This, in turn, increases the production of GDF-15 as a defense mechanism.

We also found that subjects with TPOAb positivity had lower GDF15 levels. To the best of our knowledge, no previous studies have tested the association between GDF15 and TPOAb positivity. GDF15 was reported to be a biomarker of autoimmune diseases, such as autoimmune hepatitis [36]. Specifically, GDF15 was useful to distinguish autoimmune hepatitis from other liver diseases and a recent study showed that patients with autoimmune hepatitis were GDF15 positive after immunohistological staining in the hepatic cytoplasm and sinusoidal endothelial cells [36]. The same authors also demonstrated that remission of autoimmune hepatitis was associated with reduced GDF15 levels as well as positivity in the liver tissue. Similarly, patients with rheumatoid arthritis had higher GDF15 levels, which also correlated with disease activity [37]. Patients with systemic lupus erythematosus showed higher levels of GDF5 compared to healthy controls, positively correlated with hematuria and disease activity, and was inversely associated with C3 and C4 [38]. The same study also demonstrated that treatment with GDF15 reduced renal damage and reversed the increased percentage of CD11b+ cells, thus possibly inhibiting/reducing CD11b+ cell proliferations [38]. However, the role of GDF15 in autoimmune disease is controversial, as demonstrated in type 1 diabetes, where it was shown to have a possible protective role. Indeed, the administration of GDF15 protected β cells from apoptosis induced by interferon γ and interleukin 1β and significantly decreased insulitis compared to the control group [39]. GDF15 has also been evaluated in patients with multiple sclerosis, where it represents a biomarker of a stable course of disease [40]. Overall, GDF15 seems to protect tissues against inflammation, but its role in different pathologies is context-dependent. Future studies will help to elucidate the pathophysiologic relationship between TPOAb and GDF15.

## 4. Materials and Methods

### 4.1. Study Population

The sample of this analysis comes from the SardiNIA survey, which is a population-based study that investigates thousands of genetic and phenotypic traits associated with aging. Briefly, since 2001 all residents in Lanusei, Arzana, Ilbono, and Elini (Sardinia, Italy) aged at least 14 years old were invited to participate. In all, 62% of the eligible population (n = 6148) were recruited and visited every 3–4 years, this generated 5 complete sets of visits.

For the purpose of this study, we excluded participants who self-reported use levothyroxine or thyrostatics, as well as drugs that could alter thyroid hormones (i.e., corticosteroids, amiodarone, and/or lithium), reaching a final sample of 4413 subjects.

### 4.2. Assessment of Covariates

Blood pressure was measured in the morning, with subjects in the seated position, after 5 min of a quiet resting period. The average of the second and third measurements on both the right and left arms was used in the analysis. Waist circumference, height, and weight were determined for all patients. Body Mass Index (BMI) was calculated as body weight (kg)/height (m^2^). Hypertension was defined as systolic blood pressure (SBP) ≥ 140 mmHg and/or diastolic blood pressure (DBP) ≥ 90 mmHg and/or the use of anti-hypertensive drugs. Diabetes was considered in all patients who self-reported a previous diagnosis of diabetes mellitus and/or presence of fasting glycemia ≥ 126 mg/dL and/or glycated hemoglobin (HbA1c) ≥ 6.5%. Smokers were defined as current consumers of at least one cigarette per day. Each participant signed an informed consent form. All study methods were conducted according to the principles expressed in the Declaration of Helsinki and were approved by the governing Ethics Committee, Azienda Sanitaria Locale 4.

### 4.3. Laboratory Assays

Blood venous samples were drawn between 7 and 8 a.m. after an overnight fast. Serum was immediately isolated and stored at −80 °C until use. Serum GDF-15 levels were measured by ELISA (DY957 R&D Systems). The optical density of each sample was determined using a microplate reader set to 450 nm, with wavelength readings at 570 nm subtracted from the readings at 450 nm. All measurements were higher than the lower values of standard values (from 1000 to 7.8 pg/mL). Measurements were performed on a single lot of reagents to avoid batch effects. Plasma triglycerides and total cholesterol were quantified with an enzymatic method (Abbott Laboratories ABA-200 ATC Biochromatic Analyzer, Irving, TX, USA). High-density lipoprotein cholesterol was assessed by a dextran sulfate–magnesium precipitation procedure. Low-density lipoprotein cholesterol was calculated with the Friedwald formula. Serum samples were also assayed for thyrotropin (TSH), free thyroxine (FT4), and antibodies against thyroperoxidase (TPOAb) and against thyroglobulin (TGAb) using an automated assay system (Immulite 2000, Siemens, Munich, Germany), following the manufacturer’s instructions. Normal values were TSH, 0.4–4.0 μIU/mL; FT4, 0.89–1.76 ng/dL; TPOAb, <35 IU/mL; and TGAb, <40 IU/mL.

### 4.4. Statistical Analysis

Due to the skewed distribution (Shapiro–Wilk test), data are reported as median and interquartile range. Pearson χ2 was used to analyze the differences among proportions and the Wilcoxon rank sum test was used to assess the difference between continuous variables. Multivariable linear regression analysis was conducted to detect associations between GDF15 and the covariates which were significantly associated at univariate analysis. Mann–Whitney was used to compare levels of GDF15 positivity for antibodies against the thyroid. Logistic regression analysis was used to test which variables are associated with TPOAb and TGAb positivity. A two-sided *p*-value < 0.05 indicated statistical significance in STATA 12.0.

## 5. Conclusions

Reduced thyroid function may increase levels of GDF15, as demonstrated by its positive association with TSH and inverse correlation with FT4. Further studies will need to explore whether higher GDF15 levels could have a role in the increased cardiovascular risk found in patients with hypothyroidism.

## Figures and Tables

**Table 1:** Descriptive statistics of the sample.

	Female	Male	Total
n	2429	1984	4413
Age	47.4 (35.6–60.6)	49.0 (37.1–63.3) *	48.1 (36.2–61.8)
BMI, Kg/m^2^	24.0 (21.2–27.7)	26.6 (24.1–29.2) ^#^	25.4 (22.4–28.7)
Waist, cm	81 (74–90)	92 (86–100) ^#^	87 (78–95)
TSH (mUI/mL)	1.71 (1.17–2.51)	1.40 (0.98–2.04) ^#^	1.54 (1.08–2.33)
FT4 (ng/dL)	1.06 (0.96–1.20)	1.07 (0.97–1.21)	1.06 (0.96–1.20)
GDF15, pg/mL	202.7 (156.4–277.4)	204.4 (155.4–290.5)	203.6 (155.9–281.3)
Diabetes, n (%)	104 (3.7%)	103 (5.0%) *	307 (4.3%)
Hypertension, n (%)	785 (28.1%)	804 (39.3%) ^#^	1589 (32.8%)
Smokers, n (%)	399 (14.3%)	496 (24.2%) ^#^	895 (18.5%)
TPOAb positivity, n (%)	531 (19.0%) ^#^	206 (10.1%)	737 (15.2%)
TGAb positivity, n (%)	340 (12.1%) ^#^	121 (5.9%)	461 (9.5%)

Abbreviations: TSH, thyrotropin; yrs, years; BMI, body mass index; FT4, free thyroxine; TPOAb, antibody against thyroperoxidase; TGAb, antibody against thyroglobulin; GDF15, human growth differentiation factor 15. * *p* < 0.05. ^#^ *p* < 0.001.

**Table 2:** Association of clinical and biochemical parameters with GDF15: results of the Spearman correlation analysis.

Variable	Rho	*p*
Age	0.619	<0.001
BMI	0.254	<0.001
HbA1c	0.276	<0.001
TSH	−0.108	<0.001
FT4	−0.019	<0.001
TPOAb	−0.087	0.029
Smoke	−0.048	<0.001
Total cholesterol	−0.071	<0.001
LDL	0.043	0.004
HDL	−0.034	0.028
Triglycerides	0.178	<0.001

Abbreviations: BMI, body mass index; HbA1c, glycated hemoglobin; TSH, thyrotropin; FT4, free thyroxine, LDL, low density lipoprotein; HDL, high density lipoprotein.

**Table 3:** Results of multiple regression analysis.

Variable	Beta	t	Std. Err.	*p*
Age	0.416	28.82	0.165	<0.001
TSH	0.031	2.55	1.354	0.011
FT4	−0.030	−2.25	10.580	0.024
Total cholesterol	−0.088	−6.77	0.060	<0.001
Diabetes	0.194	14.79	12.141	<0.001
Hypertension	0.041	3.06	5.345	0.002
Smoke	0.037	2.86	6.267	0.004

Abbreviations: TSH, thyrotropin; FT4, free thyroxine.

**Table 4:** Variation in GDF15 across autoantibody positivity in older subjects.

Variable	GFD15 (pg/mL)	GFD15 (pg/mL)
	Abs Standard Cut-Off ^#^	Abs High Cut-Off ^##^
TPOAb		
Negative	260.5 (201.2–192.9) *	260.6 (201.4–361.7) **
Positive	250.1 (192.9–335.8)	240.6 (176.2–314.2)
TGAb		
Negative	258.5 (200.2–360.0)	257.4 (199.5–356.7)
Positive	262.3 (196.9–330.5)	270.2 (209.3–346.0)

Abbreviations: Abs, antibodies; TPOAb, antibody against thyroperoxidase; TGAb, antibody against thyroglobulin ^#^ TPOAb ≥ 45 IU/mL; TGAb ≥ 40 IU/mL ^##^ TPOAb and TGAb ≥ 100 IU/mL; * *p* < 0.05, ** *p* < 0.001.

## Data Availability

The data generated in this study are available upon request from the corresponding author.
